# IFI44 is an immune evasion biomarker for SARS-CoV-2 and *Staphylococcus aureus* infection in patients with RA

**DOI:** 10.3389/fimmu.2022.1013322

**Published:** 2022-09-15

**Authors:** Qingcong Zheng, Du Wang, Rongjie Lin, Qi Lv, Wanming Wang

**Affiliations:** ^1^ Department of Orthopedics, 900th Hospital of Joint Logistics Support Force, Fuzhou, China; ^2^ Arthritis Clinical and Research Center, Peking University People’s Hospital, Beijing, China

**Keywords:** SARS-CoV-2, COVID-19, *Staphylococcus aureus*, Rheumatoid arthritis, IFI44, dendritic cells, 1,25-dihydroxy vitamin D_3_

## Abstract

**Background:**

Severe acute respiratory syndrome coronavirus 2 (SARS-CoV-2) caused a global pandemic of severe coronavirus disease 2019 (COVID-19). *Staphylococcus aureus* is one of the most common pathogenic bacteria in humans, rheumatoid arthritis (RA) is among the most prevalent autoimmune conditions. RA is a significant risk factor for SARS-CoV-2 and *S. aureus* infections, although the mechanism of RA and SARS-CoV-2 infection in conjunction with *S. aureus* infection has not been elucidated. The purpose of this study is to investigate the biomarkers and disease targets between RA and SARS-CoV-2 and *S. aureus* infections using bioinformatics analysis, to search for the molecular mechanisms of SARS-CoV-2 and *S. aureus* immune escape and potential drug targets in the RA population, and to provide new directions for further analysis and targeted development of clinical treatments.

**Methods:**

The RA dataset (GSE93272) and the *S. aureus* bacteremia (SAB) dataset (GSE33341) were used to obtain differentially expressed gene sets, respectively, and the common differentially expressed genes (DEGs) were determined through the intersection. Functional enrichment analysis utilizing GO, KEGG, and ClueGO methods. The PPI network was created utilizing the STRING database, and the top 10 hub genes were identified and further examined for functional enrichment using Metascape and GeneMANIA. The top 10 hub genes were intersected with the SARS-CoV-2 gene pool to identify five hub genes shared by RA, COVID-19, and SAB, and functional enrichment analysis was conducted using Metascape and GeneMANIA. Using the NetworkAnalyst platform, TF-hub gene and miRNA-hub gene networks were built for these five hub genes. The hub gene was verified utilizing GSE17755, GSE55235, and GSE13670, and its effectiveness was assessed utilizing ROC curves. CIBERSORT was applied to examine immune cell infiltration and the link between the hub gene and immune cells.

**Results:**

A total of 199 DEGs were extracted from the GSE93272 and GSE33341 datasets. KEGG analysis of enrichment pathways were NLR signaling pathway, cell membrane DNA sensing pathway, oxidative phosphorylation, and viral infection. Positive/negative regulation of the immune system, regulation of the interferon-I (IFN-I; IFN-α/β) pathway, and associated pathways of the immunological response to viruses were enriched in GO and ClueGO analyses. PPI network and Cytoscape platform identified the top 10 hub genes: RSAD2, IFIT3, GBP1, RTP4, IFI44, OAS1, IFI44L, ISG15, HERC5, and IFIT5. The pathways are mainly enriched in response to viral and bacterial infection, IFN signaling, and 1,25-dihydroxy vitamin D3. IFI44, OAS1, IFI44L, ISG15, and HERC5 are the five hub genes shared by RA, COVID-19, and SAB. The pathways are primarily enriched for response to viral and bacterial infections. The TF-hub gene network and miRNA-hub gene network identified YY1 as a key TF and hsa-mir-1-3p and hsa-mir-146a-5p as two important miRNAs related to IFI44. IFI44 was identified as a hub gene by validating GSE17755, GSE55235, and GSE13670. Immune cell infiltration analysis showed a strong positive correlation between activated dendritic cells and IFI44 expression.

**Conclusions:**

IFI144 was discovered as a shared biomarker and disease target for RA, COVID-19, and SAB by this study. IFI44 negatively regulates the IFN signaling pathway to promote viral replication and bacterial proliferation and is an important molecular target for SARS-CoV-2 and *S. aureus* immune escape in RA. Dendritic cells play an important role in this process. 1,25-Dihydroxy vitamin D3 may be an important therapeutic agent in treating RA with SARS-CoV-2 and *S. aureus* infections.

## Introduction

Rheumatoid arthritis (RA) (1) is one of the most prevalent chronic inflammatory autoimmune diseases and has been the focus of intense study for decades (1–5). The prevalence of RA is about 1% (6). The clinical presentation of RA is characterized by chronic persistent synovitis, which, in turn, destroys bone and cartilage, leading to joint bone destruction and chronic disability (7–9). Therefore, patients with RA are more prone than the general population to requiring hip and knee replacements (10). There are three main categories of factors that influence the progression of RA: genetic, environmental, and immune (11, 12), with microbial infections (e.g., bacteria and viruses) constituting a significant subset of environmental factors that can trigger, induce, and exacerbate the disease process in RA (13–17). The balance between the impact of microorganisms on the host and the immune response of the host to microorganisms is crucial for maintaining the regular functioning of the body’s immune system, and an imbalance between these reactions can exacerbate autoimmune inflammation in RA (18). In addition, disease-modifying antirheumatic drugs (DMARDs) and glucocorticoids, commonly used for RA, can affect the immune system to varying degrees (19–21). Although emerging biologic medicines (e.g., TNF inhibitors) have been employed in recent years to treat patients with RA with an inadequate response to DMARDs (22–25), studies have shown that their usage is linked with an increased risk of infection in patients with RA (26–28). Therefore, microbial infection is dangerous for individuals with RA, either in the illness itself or with the associated medicine, as well as after arthroplasty (29–31).

*Staphylococcus aureus* is a gram-positive human opportunistic pathogen (32) that frequently colonizes the human nasal cavity (33, 34) and can cause severe systemic or local infections if the immune system is compromised (35). First, *S. aureus* bacteremia (SAB) is a frequent systemic infection that is characterized by significant morbidity and mortality (36), and the majority of SAB are endogenous infections, predominantly from nasally colonized colonies (37). Second, local infections with *S. aureus* are prevalent in postoperative surgical-site infection (SSI) and prosthetic joint infection (PJI) (38, 39), which are not only the most prevalent postoperative complications (40) but also catastrophic consequences of joint replacement surgery (41, 42). According to studies, nasal carriage of *S. aureus* is also a common source of postoperative infections (43, 44). Patients with RA are more likely than the general population to carry *S. aureus* in their nasal vestibules (45), and RA medications enhance nasal *S. aureus* carriage (46). In a Danish national observational cohort study, RA was identified as a significant risk factor for SAB, and intra-articular orthopedic implants enhanced the chance of infection (31). Another prospective cohort study found that patients with RA had a greater incidence of SAB and death and that RA-induced osteoarthritic damage made *S. aureus* more vulnerable to osteoarthritic infection (47). Patients with RA are susceptible to *S. aureus* primarily due to the following factors: First, the immune system disorder of patients with RA makes *S. aureus* easy to invade the host. Second, for the local situation of patients with RA, the bone and joint damage caused by the disease makes local infection with *S. aureus* easier. Third, patients with RA are susceptible to carrying *S. aureus*. Fourth, the medication of RA makes the nasal cavity more susceptible and carries more *S. aureus*. Fifth, it is easy for *S. aureus* to cause SSI and PJI in patients with RA who have had joint replacement surgery. Therefore, we aim to investigate the RA population’s underlying susceptibility mechanism to *S. aureus*.

In 2019, COVID-19, caused by the severe acute respiratory syndrome coronavirus 2 (SARS-CoV-2), swiftly affected people and produced a significant public health concern, which was eventually classified as a worldwide pandemic (48–52). As of 12 June 2022, over 535 million confirmed cases and over six million deaths had been reported globally (53). COVID-19 is a systemic disease that can cause significant damage to several body systems, manifesting clinically as fever, cough, and respiratory distress, as well as skeletal and muscular symptoms, including arthralgia (54–57). SARS-CoV-2 has been reported to overstimulate the body’s immune system and contribute to autoantibody production due to potential antigenic cross-reactivity with the body (58–60). Indeed, patients with COVID-19 frequently exhibit immunological dysregulation (61) and can trigger multiple autoimmune diseases (59, 62), and, conversely, patients with autoimmune disease are more vulnerable to SARS-CoV-2 infection (63), and the course of COVID-19 is more severe in hospitalized patients with autoimmune disease (64). As one of the most prevalent autoimmune diseases, RA merits in-depth investigation. According to studies, patients with RA infected with SARS-CoV-2 had a greater likelihood of hospitalization and mortality than non-RA patients (65, 66). Moreover, viral sequelae/combined bacterial infections are not only common consequences (67–69) but also significantly exacerbate disease severity and death (70–74). *Streptococcus pneumoniae*, β-hemolytic streptococci, *Haemophilus influenzae*, *Pseudomonas aeruginosa*, and *S. aureus* are often coinfected microorganisms (75–80). In COVID-19, *S. aureus* was the most common bacterium for SARS-CoV-2 sequel/combination (81, 82).

The RA population is one of the most numerous in the world for autoimmune diseases, with *S. aureus* being one of the most common human pathogens and COVID-19 caused by SARS-CoV-2, leading to a global pandemic. These three factors affect a wide range of populations and have a poor prognosis, and their combination leads to high rates of disability and mortality, posing a serious risk to global public health. This study aims to investigate the causes of RA susceptibility to SARS-CoV-2 and Aureus infection through bioinformatics analysis, to discover the common biomarkers and disease targets among the three, to search for the mechanisms of SARS-CoV-2 and *S. aureus* immune escape in the RA population, and to provide new directions for further analysis of their pathogenesis and targeted development of clinical treatments.

## Materials and methods

### Data collection

Three RA (GSE93272, GSE17755, GSE55235) and two *S. aureus* infection (GSE33341 and GSE13670) datasets were included in this study (83–87), using the National Center for Biotechnology Information Gene Expression Omnibus (GEO) (https://www.ncbi.nlm.nih.gov/geo/) for screening (**Table 1**). As test sets, the GSE93272 dataset with 232 patients with RA and 43 healthy individuals’ whole blood samples and the GSE33341 dataset with 31 SAB patients and 43 healthy individuals’ whole blood samples were utilized to identify the differentially expressed genes (DEGs). The GSE17755 dataset contains whole-blood samples from four patients with RA and 12 healthy individuals. The GSE55235 dataset contains synovial tissue samples from 10 patients with RA and 10 healthy individuals. GSE13670 dataset contains 15 *S. aureus*-infected macrophage samples and 15 healthy human macrophage samples. These three datasets were utilized as validation sets for the hub genes.

**Table 1:** Basic information of selected datasets.

Dataset ID	Platform	Tissue (*Homo sapiens*)	Experimental group	Normal control	Experiment type	Reference
GSE93272	GPL570	Whole blood	232	43	Array	Tasaki et al. (83)
GSE33341	GPL571	Whole blood	31	43	Array	Ahn et al. (84)
GSE17755	GPL1291	Whole blood	4	12	Array	Lee et al. (85)
GSE55235	GPL96	Synovium	10	10	Array	Woetzel et al. (86)
GSE13670	GPL570	PBMC	15	15	Array	Kozielet al. (87)

### Identification of DEGs

The empirical Bayesian approach of the limma R package (http://www.bioconductor.org/packages/release/bioc/html/limma.html) (88, 89) was used for differentially expressed genes between the RA and HC groups of GSE93272 and the SAB and HC groups of GSE33341 for analysis. The cutoff was |log2 FC| > 0.5 and P < 0.05. The volcano map was further drawn using the ggplot2 package to reflect the differential expression of DEGs. The common DEGs were obtained by taking the intersection of DEGs (GSE93272) and DEGs (GSE33341) using the Venn-diagram package in the R software.

### GO, KEGG, and ClueGO enrichment analyses of DEGs

To explore the pathways and functions of the identified genes, GO and KEGG enrichment analyses of common-DEGs were performed with the R package “clusterProfiler” (90, 91). *P* < 0.05 indicates statistical significance. Finally, we visualized the common DEGs by using ClueGO (a plug-in for Cytoscape that uses the Kappa statistical analysis method) to display the interactive gene network map and analyze the function of the target gene set.

### PPI network analysis, machine learning, and the identification of hub genes

The STRING database (https://string-db.org/) (92) was utilized to filter and construct PPI networks based on confidence values greater than 0.40. Machine learning is used to predict the interactions of PPI networks, specifically using the k-means algorithm (network is clustered to a specified number of clusters; number of clusters: 3) for clustering. K-means is an effective unsupervised machine learning approach for predicting protein pairings that interact without prior data labeling (93, 94). We construct and visualize PPI network data using The Cytoscape platform (95), then analyze PPI molecular networks using The MCODE (a Added to abbreviations Cytoscape plug-in). The cytoHubba tool was utilized to find hub genes, analyze each gene using the maximum centrality (MCC) algorithm, rank these genes, and filter the top 10 hub genes.

### Metascape and GeneMANIA enrichment analyses of hub genes

Metascape (https://metascape.org/gp/index.html#/main/step1) is a statistical approach that can use all genes in the input genome as an enrichment background. Genes are grouped into clusters using terms with a *P*-value of 0.01, a minimum count of 3, and an enrichment factor > 1.5 to look for enrichment pathways and related functional annotations of target genes. In addition, terms with a similarity of greater than 0.30 were connected point to point by the Cytoscape visual network program to generate a network diagram that further illustrates the relationships between terms. GeneMANIA (http://www.genemania.org) is another website that integrates different databases and technologies to anticipate and identify the relevant activities of individual genes in hub genes and establish gene priority and linkages.

### Identification of hub genes between RA, COVID-19, and SAB and functional enrichment analysis

The GeneCards database (https://www.genecards.org/) (96) was accessed and searched for “COVID-19” and “SARS-CoV-2” as keywords, and 4,778 and 4,055 related genes were found, respectively. There were 17, 28, and 25 SARS-CoV-2–associated genes from Ziegler et al. (97), Jain et al. (98), and Xiong et al. (99), respectively (**Table 2**). Finally, 5,103 related genes were obtained after summarizing these five parts of genes and removing duplicate data. Hub genes were obtained from the intersection of the top 10 hub genes and 5,103 SARS-CoV-2–related genes using the Venn Diagram package in R software. Finally, Metascape and GeneMANIA enrichment analyses of the hub gene were used.

**Table 2:** SARS-CoV-2–associated genes in the relevant reference.

Reference	Tissue (*Homo sapien*s)	Experiment type	Gene ID
Ziegler et al., 2020 (97)	Nasal polyps, lung lobe, ethmoid sinus surgical tissue, ileum	Array	STAT1,IFI6,IFNAR1, IFNGR2,GBP2,IFITM1,TRIM27, NT5DC1, ARL6IP1,TMPRSS2, ACE2, TRIM28, APOA1, FABP6, ENPEP, FI35, XAF1
Jain et al., 2020 (98)	Nasopharyngeal swabs	Array	IFI44,IFIT1,IFIT1B, IFIH1,IL6, IL10, IL11, IL19, IL3RA,IL21RA,IL18R1,CXCL5, CXCL12, CCL2, CCL4, CXCL10,CSF2, TNFSF11, TNFRSF11B, BMP2, BMP7, PDGFA,C4BPA, CCR6, CCR22, CCR25, SERPINE1, SERPINF2
Xiong et al., 2020 (99)	Peripheral blood mononuclear cells, bronchoalveolar lavage fluid	Array	CXCL1, CXCL2, CXCL6, CXCL8,CXCL10, CXCL10/IP-10,CCL2/MCP-1,CCL3/MIP-1A, CCL4/MIP1B,IL 33, IL18, IL10,TNFSF10, TIMP1, C5, AREG, NRG1, ADA2, HK1, GAT1,PGD, PLA2G15, CTSD, GAA, LAIR1

### Construction of TF-hub genes and miRNA-hub gene network

TF-hub gene and miRNAs-hub gene regulation networks were constructed utilizing NetworkAnalyst (https://www.networkanalyst.ca/) (100). We submitted the hub genes between RA, COVID-19, and SAB to NetworkAnalyst to acquire TFs from the ENCODE database and miRNA from miRTarBase v8.0 and TarBase v8.0 for hub genes. Cytoscape was used to display the networks of TF-hub genes and miRNA-hub genes.

### Validation of hub genes and identification of hub genes

The datasets GSE17755, GSE55235, and GSE13670 were included in our study as validation sets to strengthen the reliability and correctness of the results. The genes from the three validation sets were also individually processed using the limma R package to generate the volcano map of the corresponding DEGs. The hub genes were identified using the Venn Diagram tool, and the expression of the hub genes in each dataset was visualized using a box plot. The subject Receiver Operating Characteristic Curve (ROC) was then used to estimate the test’s effect to determine the hub gene’s sensitivity and specificity in various datasets (101). A value of 0.7 was regarded as diagnostically significant.

### Analysis of immune cell infiltration and correlation analysis between immune cells

The CIBERSORT algorithm (http://CIBERSORT.stanford.edu/) is a method based on linear support vector regression (102). It was applied to evaluate the composition and quantity of immune cells in RA and HC. *P <* 0.05 prompted us to submit the data to CIBERSORT and receive the immune cell infiltration matrix. The ggplot2 package was used to display the distribution of LM22, whereas the corrplot package was utilized to display its correlation. Finally, we used Pearson’s correlation coefficient analysis to reveal the relationship between the expression of target genes and the abundance of immune cells in RA to find immune cells associated with the target genes. The Github page for this study is HTTPS(https://github.com/zheng5862/IFI44).

## Results

### Identification of DEGs

**Figure 1:**
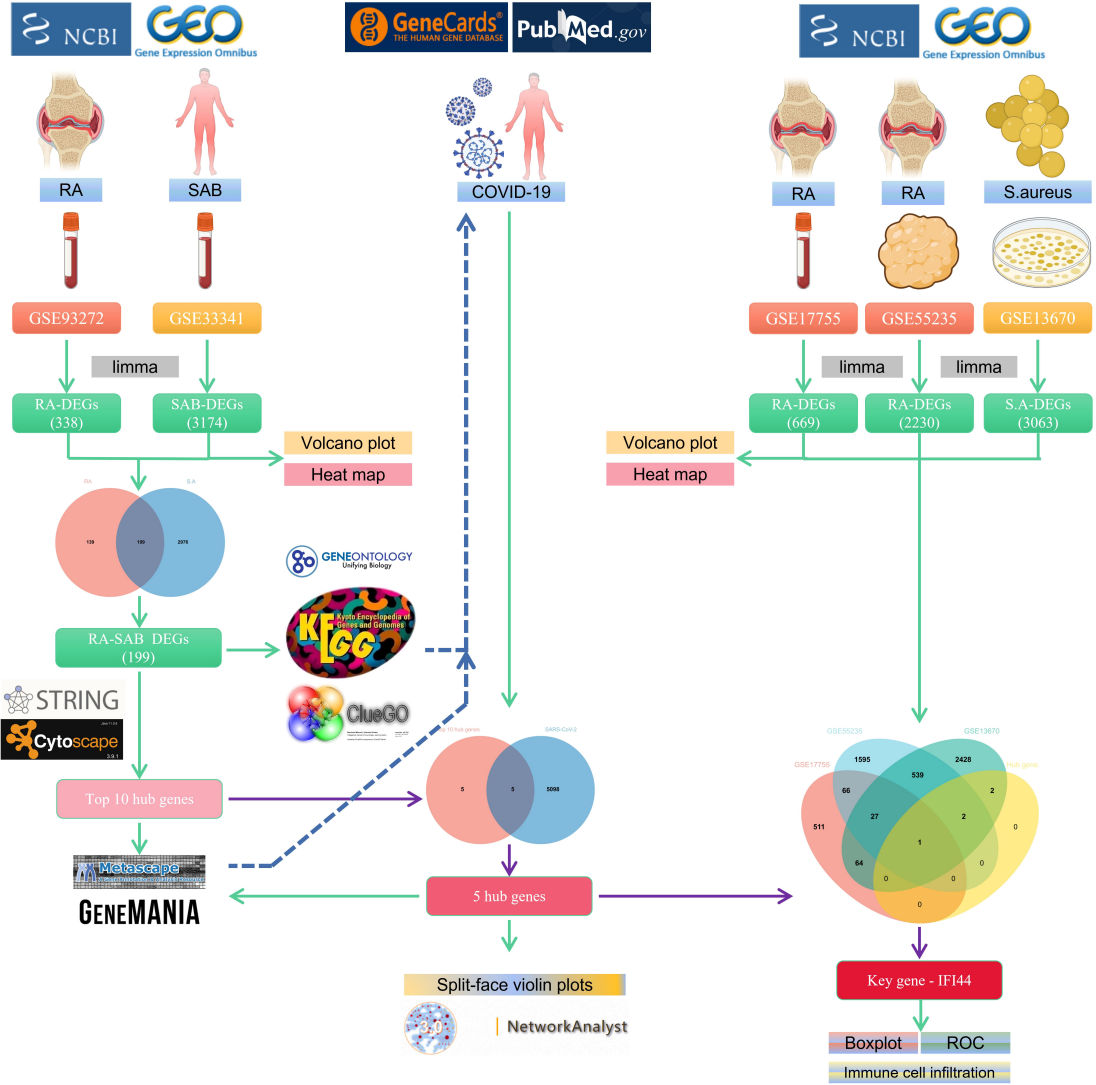
The schematic block diagram of the entire workflow of this study.

**Figure 2:**
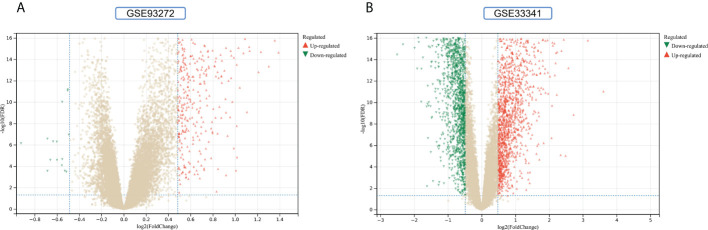
DEGs identification. **(A)** Gray dots represent genes not substantially differently expressed in RA and HC groups (*P >* 0.05), red triangles represent upregulated genes (*P <* 0.05), and green triangles represent downregulated genes (*P <* 0.05) in the GSE93272 dataset. **(B)** Gray dots represent genes not substantially differently expressed in *S. aureus* and HC groups (*P >* 0.05), red triangles represent upregulated genes (*P <* 0.05), and green triangles represent downregulated genes (*P <* 0.05) in the GSE33341 dataset.

**Figure 3:**
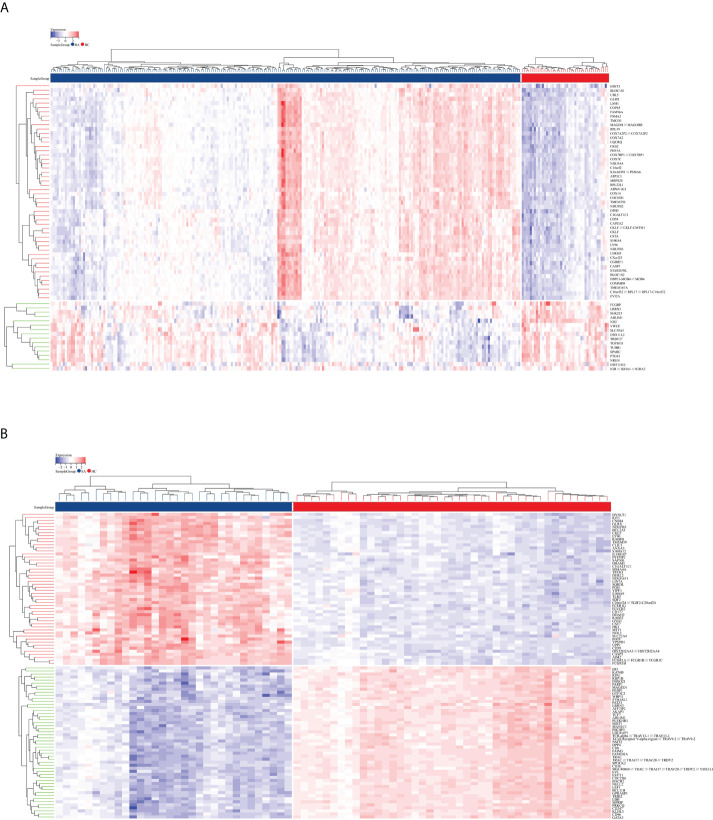
DEG distribution. **(A)** The clustering heat map shows the DEGs in the GSE93272 dataset. The RA group’s samples are colored blue, whereas the HC group’s samples are colored red. Red rectangles indicate elevated genes (*P <* 0.05), whereas blue rectangles indicate downregulated genes (*P <* 0.05). **(B)** The clustering heat map shows the intersection of DEGs in the GSE33341 dataset. The SA group’s samples are colored blue, whereas the HC group’s samples are colored red. Red rectangles indicate elevated genes (*P <* 0.05), whereas blue rectangles indicate downregulated genes (*P <* 0.05).

**Figure 4:**
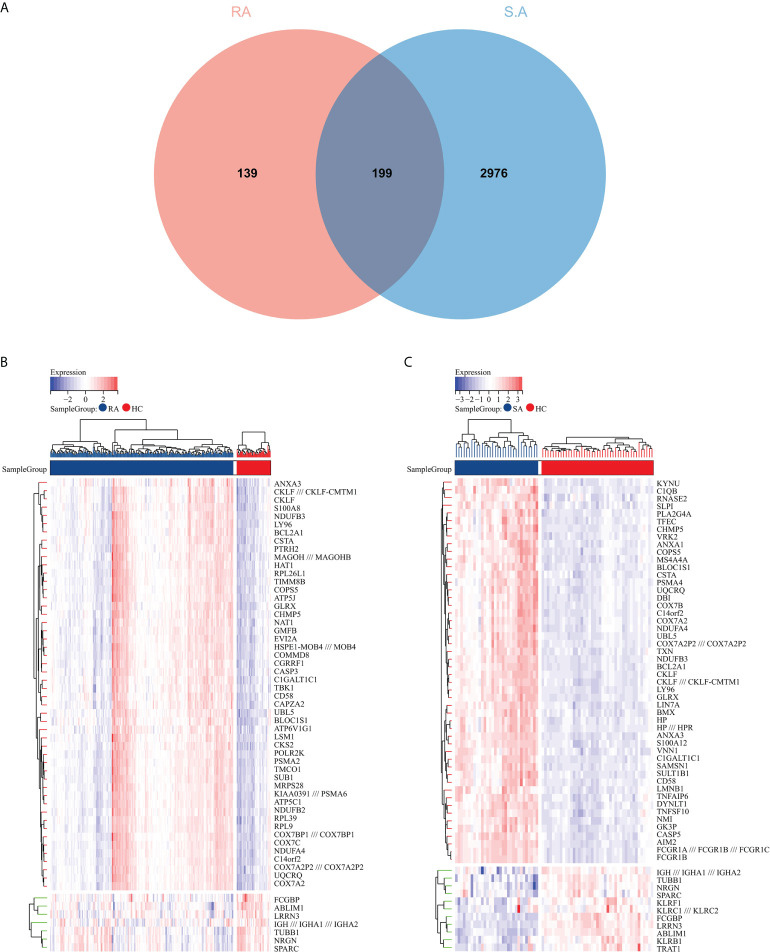
Common DEG screening. **(A)** Venn diagram on GSE93272 DEGs and GSE33341 DEGs. **(B)** Clustered heat map of common DEGs belonging to the RA group. **(C)** Clustered heat map of common DEGs in the SA group, with the RA/SA group colored blue and the HC group colored red. Red rectangles indicate elevated genes (P < 0.05), whereas blue rectangles indicate downregulated genes (P < 0.05).

The flowchart shows all our study’s key and important procedures (**Figure 1**). A total of 338 DEGs were obtained from the GSE93272 dataset, of which 322 were upregulated genes and 16 were downregulated genes. In addition, 3,174 DEGs were obtained from the GSE33341 dataset, of which 1,429 were upregulated genes and 1,745 were downregulated genes. The distribution of DEGs for the two datasets was visualized using a volcano plot (**Figure 2**) and clustered heat map analysis (**Figure 3**). The analysis results of these two datasets were intersected using the Venn Diagram package to obtain a total of 199 DEGs (**Figure 4A**). The 199 DEGs had 192 upregulated genes in GSE93272, seven downregulated genes in GSE33341, 188 upregulated genes in GSE33341, and 11 downregulated genes in GSE33341. The distribution of the 199 DEGs in the microarray datasets GSE93272 and GSE33341, respectively, can be seen using the clustered heat map (**Figures 4B,C**).

### Functional enrichment analyses of DEGs

**Figure 5:**
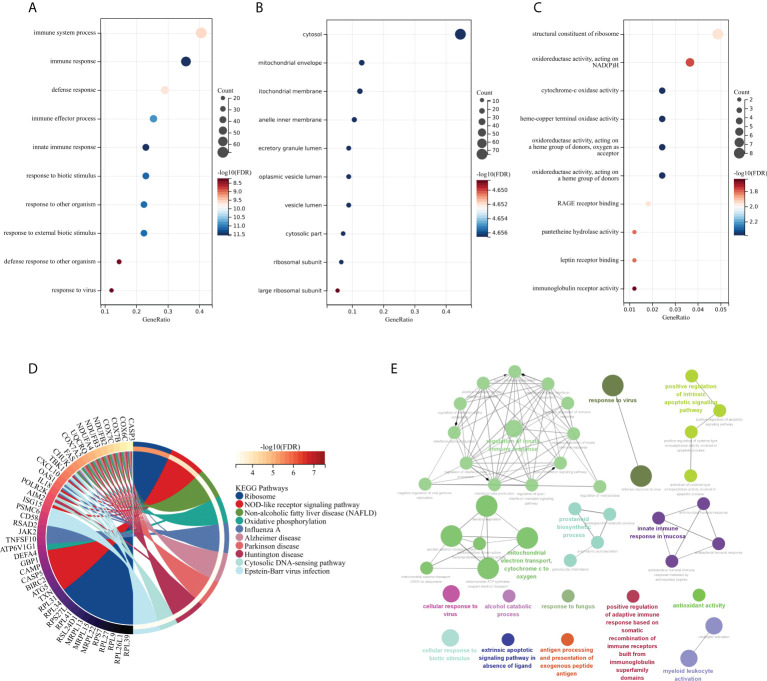
Functional enrichment analysis: GO, KEGG, and ClueGO analysis of DEGs. **(A)** BP gene enrichment of DEGs. **(B)** CC gene enrichment of DEGs. **(C)** MF gene enrichment of DEGs. **(D)** Analysis of DEGs using KEGG. **(E)** Analysis of DEGs using ClueGO.

The GO and KEGG methods were used to explore the functional correlation between the 199 DEGs sets of RA and SAB. From the GO analysis, it is clear that BP is mainly manifested in immune system process, immune response, defense response, immune effector process, innate immune response, response to biotic stimulus, response to other organisms, response to external biotic stimulus, defense response to other organism, and response to a virus (**Figure 5A**). CC is mainly enriched in the cytosol and cytosolic part (**Figure 5B**). MF mainly manifests in oxidoreductase activity, cytochrome-oxidase activity, pantetheine hydrolase activity, and immunoglobulin receptor activity (**Figure 5C**). The KEGG analysis shows the main enrichment in the NOD-like receptor signaling pathway, influenza A, oxidative phosphorylation, Epstein–Barr virus infection, and cytosolic DNA-sensing pathway (**Figure 5D**). From the ClueGO analysis, it can be visualized that the main enrichment is in the following pathways. First, regulation of innate immune responses includes IFN-I production, regulation of IFN-I production, regulation of IFN-I–mediated signaling pathway, IFN-I signaling pathway, IFN-α/β production, regulation IFN-α/β production, negative regulation of immune response, and negative regulation of innate immune response. Second, controlling viral infections involves regulating the viral replication process and immune, cellular, and defensive responses to a virus (**Figure 5E**).

### PPI network, machine learning, and the identification of top 10 hub genes

**Figure 6:**
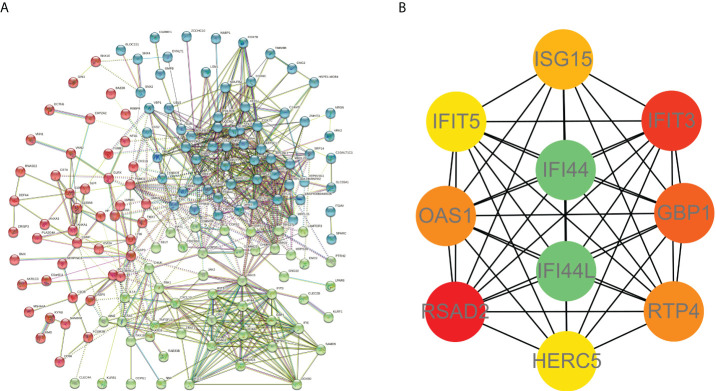
PPI interworking networks. **(A)** PPI network with 184 nodes and 750 edges. The green hexagon in the lower right corner is the top 10 hub genes derived using the CytoHubba analysis method. **(B)** Analysis of the top 10 hub genes with CytoHubba of Cytoscape.

PPI network data based on the STRING database were processed using Cytoscape software to further investigate the pathogenesis between RA and SAB. The results show that this PPI network has 184 nodes, 750 edges, an average node degree of 8.15, and an average local clustering coefficient of 0.461. The k-means cluster analysis graph based on the unsupervised machine learning algorithm of the PPI network can be seen: the green hexagon in the lower right corner is exactly the top 10 hub genes derived using the CytoHubba analysis method (**Figure 6A**). We then identified the top 10 genes in the enrichment ranking by the MCC algorithm of the CytoHubba package in Cytoscape software: RSAD2, IFIT3, GBP1, RTP4, IFI44, OAS1, IFI44L, ISG15, HERC5, and IFIT5 (**Figure 6B**), consistent with the PPI network using a k-means clustering algorithm to obtain the same results. **Tables 3**, **4** give information about the top 10 hub genes in the GSE93272 and GSE33341 datasets, respectively.

**Table 3:** Information of the top 10 hub genes in GSE93272.

Gene ID	AveExpr	Log2FC(β)	P-Value
IFI44L	8.904949446	0.918840944	1.35 × 10^−4^
ISG15	10.63569449	0.665205667	1.02 × 10^−4^
OAS1	8.99533725	0.593634568	5.15 × 10^−5^
RSAD2	9.132164541	1.008119022	4.29 × 10^−5^
GBP1	8.752490808	0.489189707	2.69 × 10^−5^
HERC5	9.587406065	0.69666656	1.19 × 10^−5^
IFI44	7.849963939	1.017400799	1.66 × 10^−6^
RTP4	7.814543847	0.590690331	3.71 × 10^−7^
IFIT3	10.88045227	0.681499851	3.43 × 10^−7^
IFIT5	8.712338631	0.698362888	1.02 × 10^−10^

**Table 4:** SARS-CoV-2-associated genes in the relevant reference.

Reference	Tissue(Homo sapiens)	Experiment type	Gene ID
Ziegler et al., 2020 (97)	Nasal polyps,Lung lobe,ethmoid sinus surgical tissue, Ileum	Array	STAT1,IFI6,IFNAR1, IFNGR2,GBP2,IFITM1,TRIM27, NT5DC1, ARL6IP1,TMPRSS2, ACE2, TRIM28, APOA1, FABP6, ENPEP, FI35, XAF1
Jain et al., 2020 (98)	Nasopharyngeal swabs	Array	IFI44,IFIT1,IFIT1B, IFIH1,IL6, IL10, IL11, IL19, IL3RA,IL21RA,IL18R1,CXCL5, CXCL12, CCL2, CCL4, CXCL10,CSF2, TNFSF11, TNFRSF11B, BMP2, BMP7, PDGFA,C4BPA, CCR6, CCR22, CCR25, SERPINE1, SERPINF2
Xiong et al., 2020 (99)	Peripheral blood mononuclear cells,Bronchoalveolar lavage fluid	Array	CXCL1, CXCL2, CXCL6, CXCL8,CXCL10, CXCL10/IP- 10,CCL2/MCP-1,CCL3/MIP-1A, CCL4/MIP1B,IL 33, IL18, IL10,TNFSF10, TIMP1, C5, AREG, NRG1, ADA2, HK1, GAT1,PGD, PLA2G15, CTSD, GAA, LAIR1

### Functional enrichment analyses of the top 10 hub genes

**Figure 7:**
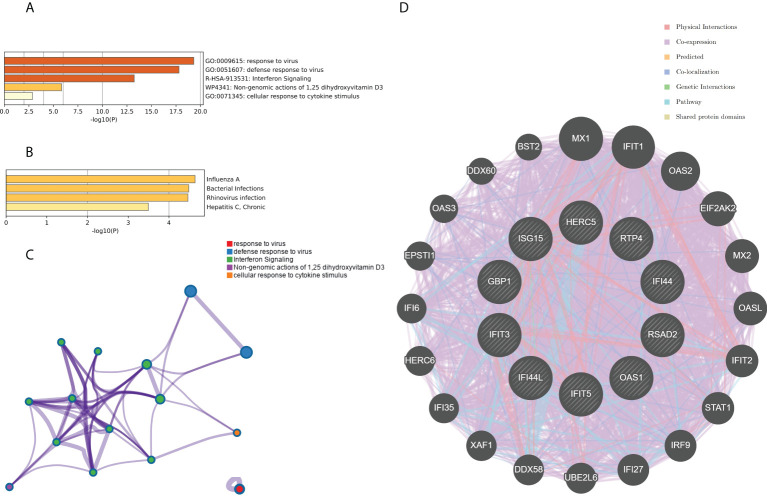
Functional enrichment analysis: Metascape and GeneMANIA of top 10 hub genes. **(A)** Pathway and process richness analysis of the Metascape platform. **(B)** Summary of enrichment analysis of DisGeNET ^13^ on Metascape platform. **(C)** The network is visualized using Cytoscape ^5^, colored by cluster IDs, and nodes sharing the same cluster ID are usually close to each other. **(D)** The gene–gene interaction network of the top 10 hub genes with the 20 most adjacent genes was analyzed using the GeneMANIA database. Each node represents a gene. The color of the linkage of the nodes represents the linkage between the corresponding genes.

The top 10 hub genes were analyzed by the Metascape platform with the following findings. First, pathway and process enrichment analysis is mainly enriched in response to a virus, defense response to a virus, interferon (IFN) signaling, non-genomic actions of 1,25-dihydroxy vitamin D3, and cellular response to cytokine stimulus (**Figure 7A**). Second, DisGeNET^13^ was mainly enriched in influenza A, bacterial infections, rhinovirus infections, and hepatitis C (chronic) (**Figure 7B**). Further network connection diagrams are used to visualize the connections between the pathways (**Figure 7C**). Finally, GeneMANIA was used to visualize the link between the 10 core genes and the most closely related genes (**Figure 7D**).

### Identification of the hub genes between RA, COVID-19, and SAB and functional enrichment analysis

**Figure 8:**
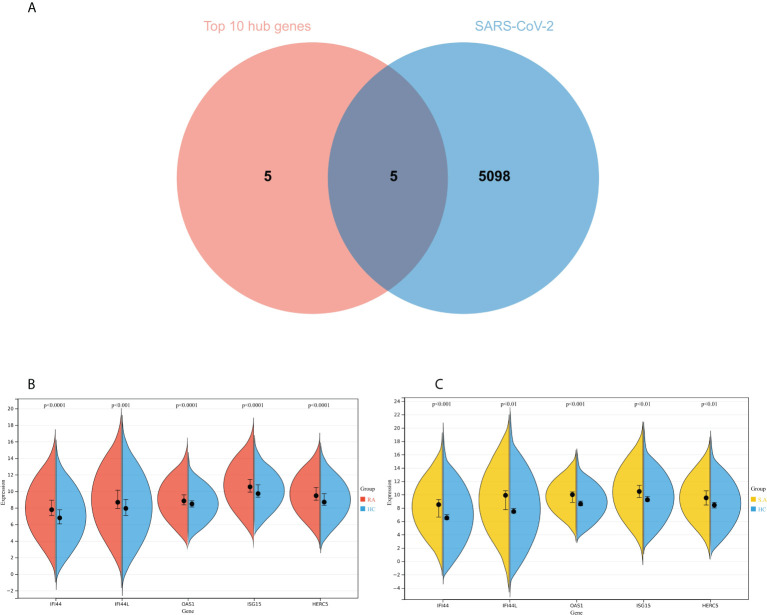
Identification of the hub gene between RA, COVID-19, and SAB. **(A)** Venn diagram of the top 10 hub genes and the SARS-CoV-2 gene set. **(B, C)** The expression of IFI44, OAS1, IFI44L, ISG15, and HERC5 in the GSE93272 and GSE33341 datasets was analyzed using split-face violin plots. Red indicates the RA group, yellow indicates the *S. aureus* group, and blue indicates the HC group.

**Figure 9:**
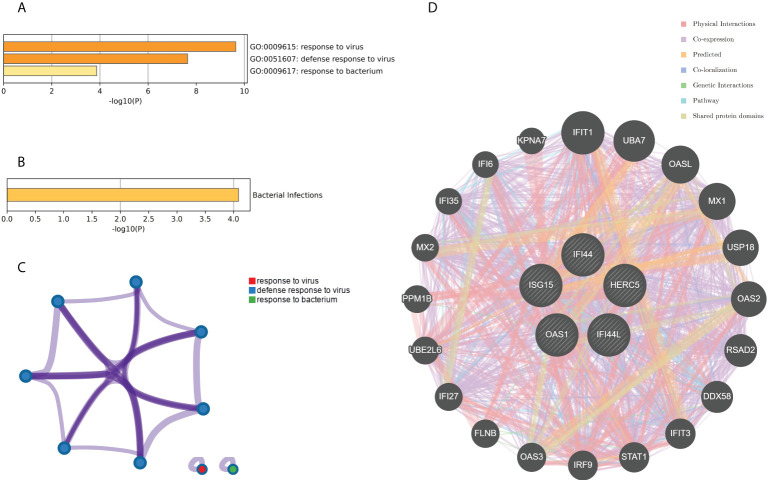
Functional enrichment analysis: Metascape and GeneMANIA of five hub genes. **(A)** Pathway and process richness analysis of the Metascape platform. **(B)** Summary of enrichment analysis of DisGeNET ^13^ on Metascape platform. **(C)** The network is visualized using Cytoscape ^5^, colored by cluster IDs, and nodes sharing the same cluster ID are usually close to each other. **(D)** The gene–gene interaction network of the five hub genes with the 20 most adjacent genes was analyzed using the GeneMANIA database. Each node represents a gene. The color of the linkage of the nodes represents the linkage between the corresponding genes.

The genes associated with SARS-CoV-2 were selected from the Genecard database and related literature, and 5,103 genes were obtained after summarizing and removing duplicate data. The top 10 hub genes intersected with the SARS-CoV-2 gene set with five genes: IFI44, OAS1, IFI44L, ISG15, and HERC5 (**Figure 8A**). The expression of these five genes in the GSE93272 and GSE33341 datasets was analyzed using split-face violin plots, and it can be seen that the expression of all five genes in the RA and SAB datasets was significantly higher than that in the control group (*P <* 0.01) (**Figures 8B, C**). The functional enrichment analysis results using the Metascape platform are as follows. First, pathway and process enrichment analysis is mainly enriched in response to a virus, defense response to a virus, and response to a bacterium (**Figure 9A**). Second, DisGeNET^13^ was mainly enriched in bacterial infections (**Figure 9B**). Further network connection diagrams are utilized to more precisely depict the links between the channels (**Figure 9C**). Finally, GeneMANIA was utilized to illustrate the relationship between the five hub genes and their closest relatives (**Figure 9D**).

### Analyses of the network of TF-hub genes and miRNA-hub genes

**Figure 10:**
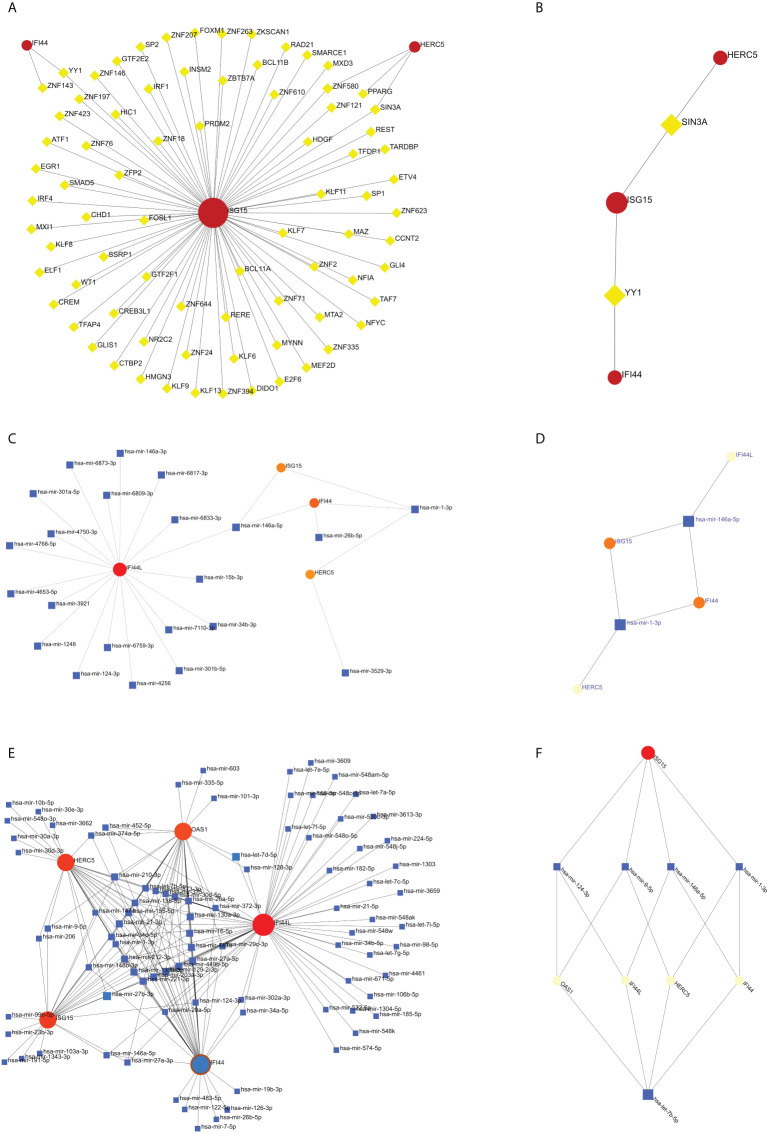
Construction of TF-hub gene and miRNA-hub gene network using NetworkAnalyst. **(A, B)** TF-hub gene network and simplified diagram. Red circles are genes, and yellow squares are TF. **(C, D)** miRNA-hub gene network (miRTarBase v8.0) and simplified diagram. **(E, F)** miRNA-hub gene network (TarBase v8.0) and simplified diagram. Circles are genes, and squares are miRNAs.

The TF of five hub genes was predicted using the ENCODE database and the NetworkAnalyst web tool. The miRNAs of five hub genes were analyzed using the miRTarBase v8.0 package and the TarBase v8.0 package of the NetworkAnalyst web tool to build the networks of TF-hub genes and miRNA-hub genes, respectively. The TF-hub gene network includes three seeds, 81 edges, and 81 nodes (**Figure 10A**), and the simplified minimum network includes three seeds, four edges, and five nodes (**Figure 10B**). YY1 has the potential to regulate ISG15 and IFI44, and SIN3A and ZNF580 have the potential to regulate ISG15 and HERC5. The network structure of miRNA-hub genes analyzed using the miRTarBase v8.0 package includes four seeds, 26 edges, and 26 nodes (**Figure 10C**). The simplified minimum network includes four seeds, six edges, and six nodes (**Figure 10D**). The network structure of miRNA-hub genes analyzed by the TarBase v8.0 package includes five seeds, 188 edges, and 94 nodes (**Figure 10E**). The simplified minimum network includes five seeds, 16 edges, and 10 nodes (**Figure 10F**). The intersection of these two miRNA-hub gene networks could reveal that hsa-mir-1-3p and hsa-mir-146a-5p may play an important role in the expression of IFI44.

### Validation of hub genes

**Figure 11:**
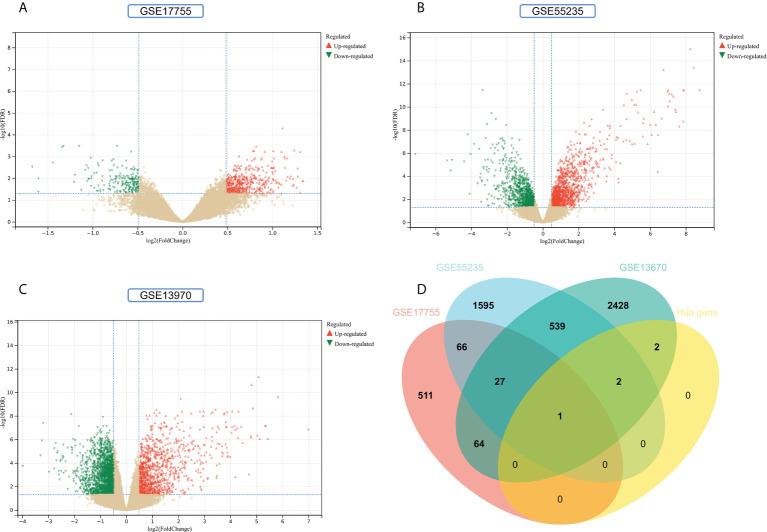
Screening for key genes. **(A, B)** Gray dots represent genes not substantially differently expressed in RA and HC groups (*P >* 0.05), red triangles represent upregulated genes (*P* < 0.05), and green triangles represent downregulated genes (*P* < 0.05) in GSE17755 and GSE55235 datasets. **(C)** Gray dots represent genes not substantially differently expressed in *S. aureus* and HC groups (*P* > 0.05), red triangles represent upregulated genes (P < 0.05), and green triangles represent downregulated genes (*P* < 0.05) in the GSE13670 dataset. **(D)** The Venn diagram of five hub genes with the three validation sets of DEGs.

**Figure 12:**
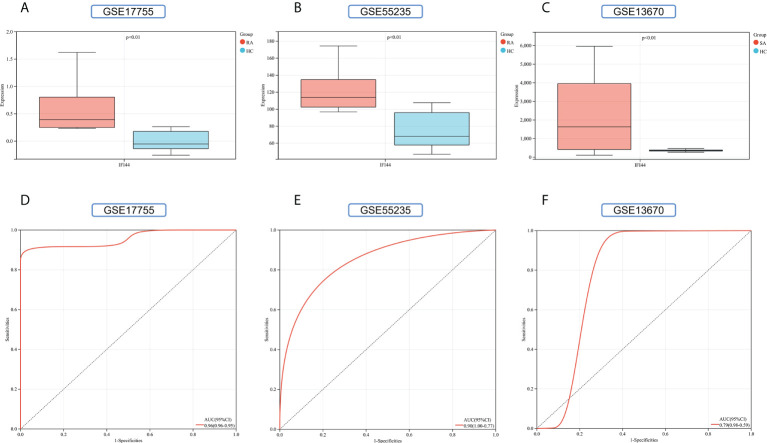
Validation of key genes. **(A–C)** The expression of IFI44 in GSE17755, GSE55235, and GSE13670. Red for RA/*S. aureus* group, and cyan for HC group. **(D–F)** The AUC of the ROC curve verifies the diagnostic validity of IFI44 in GSE17755, GSE55235, and GSE13670 (*P* < 0.05).

To improve the reliability and reproducibility of the results, we used the datasets GSE17755, GSE55235, and GSE13670 for validation. A total of 669 DEGs were obtained from the GSE17755 dataset, of which 471 were upregulated genes and 198 were downregulated genes. In addition, 2,230 DEGs were obtained from the GSE55235 dataset, of which 1,279 were upregulated genes and 951 were downregulated genes. A total of 3063 DEGs were obtained from the GSE13670 dataset, of which 1,100 upregulated genes and 1,963 downregulated genes were used. The distribution of DEGs in these three datasets was visualized using a volcano map, respectively (**Figures 11A–C**). The Venn diagram of five hub genes with the three validation sets of DEGs shows that IFI44 is the only intersection result (**Figure 11D**). IFI44 was highly expressed in all three validation sets (*P <* 0.01) (**Figures 12A–C**). Finally, the diagnostic validity of IFI44 as a biomarker was verified by ROC curves, which showed that the AUC values of IFI44 on the datasets GSE17755, GSE55235, and GSE13670 were 0.96 (95% CI, 0.95–0.96), 0.90 (95% CI, 0.77–1.00), and 0.79 (95% CI, 0.59–0.98). All had high sensitivity and high specificity (**Figures 12D–F**).

### Immune infiltration analysis

**Figure 13:**
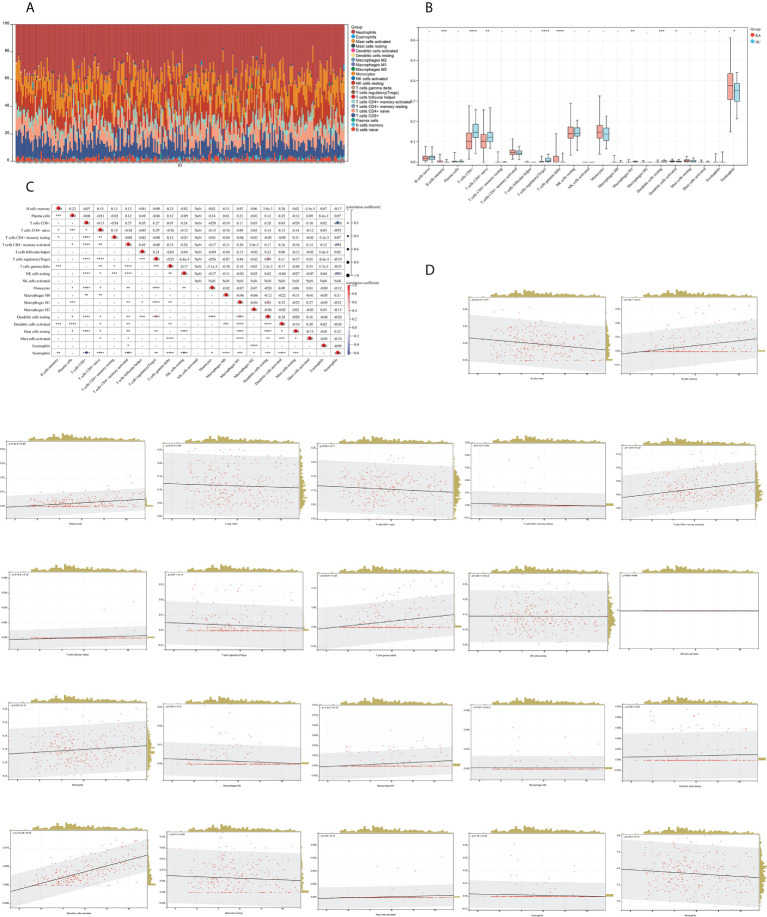
Analysis of immune cell infiltration. **(A)** A histogram of the proportion of LM22 in RA samples is depicted using the CIBERSORT algorithm, with the horizontal coordinate representing the sample and the vertical coordinate representing the percentage of individual immune cells. **(B)** Comparison of immune infiltrating cells between the RA and HC groups; red represents RA and cyan represents HC. **(C)** Correlation matrix between immune cells within the RA group. The horizontal and vertical coordinates are LM22, with red representing positive correlations and blue representing negative correlations (*P < 0.05, ** *P* < 0.01, and *** *P* < 0.001). **(D)** Correlation analysis between the expression of IFI44 and LM22.

We mapped 22 immune cell proportions in RA samples using CIBERSORT (**Figure 13A**) and then analyzed the differences in immune cell infiltration between RA and HC using box plots (**Figure 13B**). The results indicated that RA enriched four types of immune cells: B-cell memory, T-cell gamma delta, activated dendritic cells (DCs), and neutrophils (P < 0.05). Further correlation matrix analysis revealed that activated DCs were positively correlated with B-cell memory and T-cell gamma delta and negatively correlated with neutrophils (P < 0.05) (**Figure 13C**). Finally, we revealed the relationship between the expression of IFI44 and the abundance of immune cells in RA by Pearson’s correlation coefficient analysis (**Figure 13D**), which showed that only activated DCs were closely and positively correlated with IFI44 (R = 0.68, P = 3.7e-39), and activated DCs were highly enriched in RA. Thus, IFI44 may be involved in RA progression by regulating immune cell infiltration, and activated DCs may play an important role in this regard.

## Discussion

In this study, a total of 199 DEGs were obtained using a dataset of whole blood samples from RA and SAB (GSE93272 and GSE33341), and they were found to be closely associated with positive/negative regulation of the immune system and regulation of the IFN-I (IFN-α/β) pathway and related pathways of the immune system response to a virus by KEGG, GO, and ClueGO analyses. Ten hub genes were obtained using the PPI network and Cytoscape platform: RSAD2, IFIT3, GBP1, RTP4, IFI44, OAS1, IFI44L, ISG15, HERC5, and IFIT5, which were analyzed by Metascape platform and found to be associated with IFN signaling regulation and immune system response to viral infection and bacterial infection and were closely related. Five hub genes shared by RA, COVID-19, and SAB were IFI44, OAS1, IFI44L, ISG15, and HERC5, and they were found to be closely associated with the immune system response to viral infection and bacterial infection using Metascape analysis. TF-hub gene network and miRNA-hub gene network was constructed for these five hub genes, and one important TF (YY1) and two important miRNAs (hsa-mir-1-3p and hsa-mir-146a-5p) associated with IFI44 were obtained. To verify the reliability and comprehensiveness of the results, not only whole blood samples from RA (GSE17755) but also synovial tissue samples from RA (GSE55235) and blood samples from *S. aureus*-infected human mononuclear cells (GSE13670) were used to validate a core gene, which was obtained as IFI44. IFI44 was highly expressed in all five datasets, and its test efficacy was verified using ROC. Immune infiltration analysis reveals that the immune cells closely associated with IFI44 are activated DCs, which may play a significant connection between RA, SARS-CoV-2, and *S. aureus* infection. The pathway enrichment analysis revealed that 1,25-dihydroxy vitamin D3 might be an effective therapeutic agent for RA’s SARS-CoV-2 and *S. aureus* infections.

### Association of this study with The IFN-ISG pathways

IFNs are a family of cytokines having pleiotropic effects in humans (103, 104)—first recognized by Isaacs and Lindenmann in 1957 (105, 106) and characterized as antiviral inhibitors (107, 108). After more than 50 years of research by biologists, it was discovered that IFN is an essential regulator of the body’s immune system (109), which plays a crucial role not only in viral infections (110–112) but also in bacterial infections (113, 114) and autoimmune illnesses (115, 116). There are three types of IFNs: IFN-I (IFN-α, β, ϵ, κ, and ω), IFN-II (IFN-γ), and IFN-III (IFN-λ), with IFN signaling through the Janus kinase (JAK)/STAT pathway (117).

Pattern recognition receptors (PRRs) recognize pathogen-associated molecular patterns (PAMPs) (118–120) and can activate transcription factors like IRF-3 and IRF-7, as well as the NF-κB pathway of B cells (121–124). NOD-like receptors are among the important PRRs that initiate the IFN pathway. TBK-1 and IKKϵ phosphorylate IRF-3 and IRF-7 to stimulate the transcription of IFN and proinflammatory genes (125, 126), with STING serving as the upstream signaling molecule that recruits TBK-1 and IKK (127). cGAS is an important cytosolic DNA sensing that can induce IFN formation by generating the cGAMP pathway that activates STING to form the cGAS-STING pathway (128–130). Activation of the cGAS-STING pathway is a double-edged sword that plays not only a crucial function in fighting viruses (131, 132) and bacteria (133, 134) but also an aberrant activation of cGAS by its DNA, which can provoke autoimmune disorders (135). The NOD-like receptor signaling pathway, oxidative phosphorylation, and cytosolic DNA sensing in the KEGG pathway of the intersecting genes of RA and SAB in this study are reflected in the IFN pathway. In cells that are not activated by the signal, NF-κB is prevented in the cytoplasm by IκBs, and only when IκBs are phosphorylated and hydrolyzed by proteases does NF-κB migrate to the nucleus to induce the production of IFN and proinflammatory genes (136, 137). IKK is responsible for the phosphorylation of IκBs, and it consists of two kinase subunits (IKKα and IKKβ) and one regulatory subunit (IKKγ) (138, 139). Notably, FKBP5 activates IKKϵ (140), interacts with the three subunits of IKK, and promotes IKK synthesis, leading to phosphorylation of IκBs, activation of NF-κB, and its migration into the nucleus, which eventually initiates the IFN signaling pathway (141, 142).

The receptor that binds IFN-I is composed of IFNAR1 and IFNAR2 subunits (143–145), whereas the receptor that binds IFN-III is composed of IFNLR1 and IL-10R subunits (146–148). By interacting with the receptor, IFN activates JAK1 and tyrosine kinase 2 (TYK2) (149–151). Activated JAK1 and TYK2 phosphorylate and activate STAT1 and STAT2 (152–154), whereas active STAT1 and STAT2 recruit and bind IRF-9 to form ISGF3 (155, 156). The ISGF3 complex can move from the cytoplasm to the nucleus and bind to the ISRE region in the ISG promoter, thereby beginning ISG transcription (143, 157, 158). ISGs influence cell activation and death in addition to viral aspects (159), and the antiviral, antiproliferative, and immunological stress actions of ISGs allow cells and organisms to survive (160). Although IFN-I and IFN-III produce ISGs through the same mechanism, the IFN-I pathway can induce ISG expression earlier, more swiftly, and more efficiently (157, 161), and IFN-I has been the subject of most studies, triggering the production of more than 300 ISGs (162). In this study, ClueGO was used to analyze the intersectional gene enrichment pathways of RA and SAB in IFN-I production, regulation of IFN-I production, regulation of IFN-I–mediated signaling pathway, IFN-I signaling pathway, IFN-α/β production, regulation IFN-α/β production, negative regulation of immune response, negative regulation of innate immune response, regulation of viral replication process, response to a virus, defense response to viruses, and cellular response to a virus, which is reflected in the IFN-I pathway.

### IFN, ISG, and IFI44 in RA

On the basis of the findings of this study, a portion of the route of the top 10 hub genes of RA and SAB was enriched in IFN signaling and cellular response to cytokine stimulus; therefore, the association between RA and the IFN signaling pathway piqued our interest. Studies have shown that enhanced autoimmune responses can be detected in the presence of disease treated with IFN-α (163) and that 34% of patients have elevated rheumatoid factors (164), and that IFN-α treatment can contribute to the progression of RA (165, 166). In addition, the use of IFN-β1 in the treatment of MS promotes the development of RA (167). In contrast, TNF, a key driver of RA, enhances mtDNA release and initiates a cGAS/STING-dependent IFN response in inflammatory arthritis (168), and prolonged TNF therapy induces the creation of high quantities of IFN-I *via* a mechanism that stimulates IRF1 and IRF3 (169, 170). It has also been shown that significant amounts of IFN-I can be discovered in the peripheral blood of both patients with preclinical and clinical RA (115) and the synovial fluid of patients with RA (171). In reality, it dates back to 1979, when it was discovered that IFN levels were elevated in individuals with AID and positively linked with the disease’s activity (172). The possible reason for this is that PAMPs are recognized by PRRs that produce IFN-I. These PRRs include TLR, RLR, and cGAS receptors that can sense nucleic acids (173, 174).

Interestingly, these PRRs can recognize viral nucleic acids and their nucleic acids to trigger AID (175). RA is one of the most common AIDs, and IFN-I plays an important role in contributing to the development of RA (115, 176). Furthermore, IFN-I can be used as an RA biomarker and a predictor of disease progression in patients with RA (177). Recent investigations have identified a significant expression of IFN-I–induced ISGs in the peripheral blood of patients with RA (176), and this elevated expression of ISGs induced by the IFN-I signaling pathway is referred to as the IFN signature of RA (178). In peripheral blood (179, 180) and synovial fluid of patients with RA (171, 176), elevated amounts of ISGs were found. Although patients with RA correlate unequally with IFN-I and ISGs (181), IFN-I and ISGs play a role in RA susceptibility (177), and thus, IFN and ISGs are considered biomarkers and disease targets for RA (179, 182, 183).

In combating pathogenic infections, many ISGs act directly on the signaling pathways of the pathogen’s life cycle to inhibit its proliferation (158, 184). However, in RA, the excessive innate immune response and signaling dysregulation produce large amounts of IFNs that damage the organism (185). IFN desensitization is, therefore, of particular importance (158). The first aspect is cell intrinsic, which reduces signaling by blocking the JAK-STAT pathway *via* endocytosis and turnover of IFN receptors (186–189). The second aspect is that, during the immune response, some ISGs function as negative feedback regulators to maintain cellular homeostasis (158, 190, 191), and some ISGs can act as inhibitory proteins to reduce IFN pathway transduction (192). Common ISGs with negative regulatory functions include SOCS and USP18. Increased SOCS protein levels decrease the sensitivity of the JAK-STAT system, whose mechanism of action is to suppress JAK activity by binding to IFN receptors and tyrosine residues on JAK, thus preventing STAT-1 activation (193). By binding to the IFN-I receptor, USP18 can also prevent JAK activation and induce IFN-I desensitization (194). In addition, it was reported for the first time in 2019 that IFI44 also functions as a negative regulator of the IFN signaling pathway and that IFN-α treatment induces high expression of IFI44 (195) and also triggers the development of RA (196), which corresponds to our study’s finding of high expression of IFI44 in patients with RA.

IFI44 is one of the IFN-I–induced ISGs (197, 198), which was initially found in hepatitis C virus–associated microtubule aggregation protein isolation (199). Therefore, we also observed hepatitis C (chronic) pathway enrichment in the top 10 hub genes of RA versus SAB. IFI44, with the assistance of FKBP5, is capable of exhibiting the two actions listed below. First, IFI44 significantly decreases the kinase activity of IKKβ, which inhibits the phosphorylation of IκBs, which, in turn, limits NF-κB activation and restricts its migration into the nucleus (200). Second, IFI44 can reduce the kinase activity of IKKϵ, resulting in the inhibition of IRF-3 phosphorylation (125), the restriction of STAT1 phosphorylation, and the reduction of ISG production (153). The reason for the high expression of IFI44 in patients with RA is that the high expression of IFNs and ISGs in patients with RA leads to an increase in the expression of IFI44 as an ISG, and it is the negative feedback regulation of IFI44 that makes its expression significantly higher than that of the healthy population. In the results of this study, a portion of the pathways of the top 10 hub genes of RA and SAB were enriched in immune responses to viral and bacterial infections. A portion of the pathways of the top five hub genes of RA, SAB, and COVID-19 was also enriched in immune responses to viral and bacterial infections. Therefore, we followed this thought regarding the IFN pathway and continued exploring the relationship between RA, SAB, and COVID-19.

### Crosstalk between RA and SAB in terms of IFN, ISG, and IFI44

The average life expectancy of the RA population is reported to be shortened by 8 to 15 years, with infections, cardiovascular disease, and kidney disease being the three leading reasons (201–203). *S. aureus* seems inseparable from the topic of infection in patients with RA, as studies from the 1950s indicate that patients with RA are at a significantly increased risk of infection with *S. aureus* (201) and that invasion of patients with RA by *S. aureus* can result in severe deep bone and joint infections, as well as high rates of disability and mortality (47, 204). IFN-I has a crucial role in bacterial invasion of the host (205, 206), which can be both useful and damaging to the organism (207, 208), depending on the type of invading bacteria and the organism’s regulatory mechanisms (113, 209). IFN-I generated by *S. aureus* exacerbates the recruitment of leukocytes and the release of inflammatory cytokines, with detrimental effects on the organism (210–212). Because RA is an autoimmune disease capable of producing large levels of cytokines such as IFNs and ISGs, the relationship between RA and SAB *via* the IFN-I pathway can be described as follows.

On the one hand, the following points are of interest from the perspective of IFN-I–positive signaling. First, the TLR9 receptor identifies the DNA of *S. aureus*, causing DCs to produce IFN-I (213). Second, *S. aureus* detects TLR9-IRF1 *via* the Xr domain of SpA to activate the JAK-STAT pathway and NF-κB signaling pathway, resulting in the production of inflammatory cytokines such as TNF and IL-6, which promote inflammation and contribute to the progression of RA (211). Third, the autolysis process of *S. aureus* that produces peptidoglycan, among others, activates the NOD2/IRF5 pathway of DCs to mediate the IFN-I pathway, which enhances the virulence of *S. aureus* in the host to increase bacterial pathogenicity and also over-recruits neutrophils to promote inflammatory responses (210). Therefore, when patients with RA are infected with *S. aureus*, it leads to a severe proinflammatory response, probably because the superposition of the two proinflammatory mechanisms leads to an excessive inflammatory response and a severe imbalance in the immune system, followed by a collapse of the immune system, leading to a decrease in the body’s defenses and further aggravating the *S. aureus* infection, thus creating a vicious circle. On the other hand, examining the issue from the standpoint of ISGs with a negative feedback regulatory effect on the IFN-I pathway yields the following conclusions. First, SOCS has a pro-bacterial effect because it makes it easier for *S. aureus* to invade an organism’s defenses (214). SOCS not only inhibits the MYD88 molecule in macrophages to affect their antimicrobial effect (215, 216) but also inhibits the NF-κB pathway to reduce TNF release to act as an inhibitor of inflammation, thereby causing problems for host clearance of *S. aureus* (217), and an increase in phagocytosis and killing of *S. aureus* by the organism is observed when SOCS is inhibited (214). Second, USP18 can boost the susceptibility of *S. aureus* by negatively regulating the IFN-I pathway to reduce TNF-α signaling, and inhibition of USP18 can improve the body’s bacterial infection status (218).

SOCS and USP18 proteins have been reported to promote bacterial infection, whereas few IFI44 proteins have been studied. In our study, IFI44 was found to be a key crosstalk gene between RA and SAB, and IFI44 is also an IFN-I–negative regulator, which can give a decrease in antimicrobial inflammatory factors by negatively regulating the NF-κB pathway and can also inhibit STAT1 activation from blocking the production of IFN-I and ISGs (195). Thus, IFI44 may also potentially promote RA susceptibility to *S. aureus*. Many studies have suggested that the IFN-I pathway acts as a paradoxical immune response during bacterial infection of the host (218), which may be due to the different focus of the IFN-I pathway on the different stages of bacterial infection. The high expression of IFI44 protein in patients with early RA facilitates further invasion of the organism by *S. aureus*, which is one of the reasons for *S. aureus* susceptibility, and the vicious cycle of immune imbalance in the organism resulting from the excessive IFN-I cascade response prompted by late RA and *S. aureus* stimulation is one of the reasons for the poor prognosis and high mortality. We, therefore, suggest that the negative regulation of the IFN-I pathway by IFI44 expression may be one of the mechanisms of immune escape from *S. aureus*. However, most of the functions of IFI44 are unknown, and further investigation of its mechanisms in bacterial infection is a direction of interest.

### Crosstalk between RA, COVID-19, and SAB in terms of IFN, ISG, and IFI44

The coronavirus class is typically characterized by pandemic transmission and high pathogenicity; SARS-COV-2 is the ninth coronavirus identified as a severe threat to human health in 2019 (219–221). SARS-COV-2 is an enveloped virus of the genus *Betacoronavirus* with a positive-stranded single-stranded RNA genome of 26–32 kb in length (222–225). A virus is divided into four genera: α-, β-, δ-, and γ-CoV, characterized by high mutation rates and diverse recombination rates (226–229), and from 2019 to November 2021, the World Health Organization (WHO) has published Alpha (B.1.1.7), Beta (B.1.351), Gamma (P.1), Delta (B.1.617.2), and Omicron (B.1.1.529) for a total of five variants of concern (VOCs) (230). RA is associated with COVID-19 in the following points. On the one hand, SARS-CoV-2 can overstimulate the body’s immune system and has the potential for antigenic cross-reactivity with the body to trigger the creation of autoantibodies (58, 60, 62). Thus, SARS-CoV-2 infection is considered a trigger for autoimmune disease and results in a worse prognosis (231–235). On the other hand, studies indicate that patients with rheumatic disorders are at a larger risk of SARS-COV-2 infection than the general population, with a worse prognosis and increased mortality (236, 237). In the COVID-19 Global Rheumatology Alliance (C19-GRA) Global Registry and other studies, the most common rheumatic disease among patients with COVID-19 was RA (238–241). Therefore, we prefer to propose that SARS-CoV-2 infection triggers the progression of RA, that patients with RA are more susceptible to SARS-CoV-2 infection, and that the crosstalk between the two results in a vicious cycle of mutual disease progression that increases the risk of hospitalization and death (242–244), and that the crosstalk mechanism cannot be separated from the immune system and related inflammatory pathways (245).

In addition, COVID-19 combined/secondary *S. aureus* infection results in a considerable increase in mortality (246) primarily due to the following factors. First, patients with COVID-19 on admission had fewer coinfections with bacteria (3.5%) due to preventive administration of antibiotics, the most prevalent of which was *S. aureus* (81, 247–250). Second, in literature comprising 10 studies, a total of 132 bacterial species were reported as coinfections/secondary infections in patients with COVID-19 after admission, with *S. aureus* being the most common (n = 41.31%) (251). Third, according to a French study, 28% of critically ill COVID-19 patients admitted to the ICU had coinfections with bacteria, primarily *S. aureus* (252). We list a portion of the relevant literatures between RA, SAB, and COVID-19 (**Table 5**). In another bioinformatics investigation, *S. aureus* infection was shown to be the second highest in the KEGG analysis pathway enrichment order table for RA and COVID-19 (253), a result that was confirmed in our work, suggesting that there may be a connection between the IFN-I pathway in RA, COVID-19, and SAB.

**Table 5:** Literature related to coinfection between RA, SAB, and COVID-19.

Reference	Disease	Coinfection	Conclusion
Dieperink et al., 2022 (31)	RA	*S. aureus*	RA is a high risk for SAB, and orthopedic implants increase the risk.
Joost et al., 2017 (47)	RA	*S. aureus*	Patients with RA exhibit a complex course of SAB and high mortality, and RA causes a significantly increased risk of leading to OAI.
Garcia-Vidal et al., 2021 (81)	COVID-19	*S. aureus*	Coinfection at COVID-19 diagnosis was mainly *S. aureus*.
Hughes et al., 2020 (82)	COVID-19	*S. aureus*	The most common co-infecting pathogen in early COVID-19 patients is *S. aureus*.
Conway et al., 2022 (236)	RA	SARS-CoV-2	Patients with RA have higher rates of SARS-CoV-2 infection and higher mortality.
Akiyama et al., 2021 (237)	RA	SARS-CoV-2	Patients with RA are at increased risk of contracting COVID-19.

IFN-I is among the most effective cytokines secreted by the organism against SARS-CoV-2 (254, 255). However, it is not always protective for the organism. In the late stage of COVID-19, the continual strong expression of IFN-I causes inflammatory damage to the immune system and many organs, increasing the organism’s burden (256–258). It is undeniable that the IFN-I pathway had an important role in antagonizing the early stages of COVID-19 infection by secreting ISGs during the SARS-CoV-2 invasion (259, 260). However, the ISGs are not the only antiviral factors. Although most ISGs encode proteins capable of inhibiting different stages of the SARS-CoV-2 replication cycle (143, 261, 262), a few ISGs, including SOCS, USP18, and IFI44, can promote viral infection of the host (263–268). It was shown that silencing of IFI44 inhibits viral replication and overexpression of IFI44 promotes viral production due to negative regulation of the IFN-I pathway by IFI44 (195). Viruses mentioned in this study are not limited to SeV, LCMV, VSV, and IAV. Therefore, we suggest that the negative regulation of the IFN-I pathway by the expression of IFI44 may be one of the mechanisms of SARS-CoV-2 immune escape.

High expression of IFN-α in RA contributed to elevated levels of IFI44, promoted viral replication during the early stages of SARS-CoV-2 invasion, and increased susceptibility of *S. aureus*. Therefore, IFI44 may be an important target for the immune escape of SARS-CoV-2 and *S. aureus* infection in RA. Of course, we still need basic experiments and clinical trials to validate the results of our bioinformatics analysis.

### 1,25(OH)_2_VD_3_ may be an effective therapeutic agent in treating RA with SARS-CoV-2 and *S. aureus* infections

**Figure 14:**
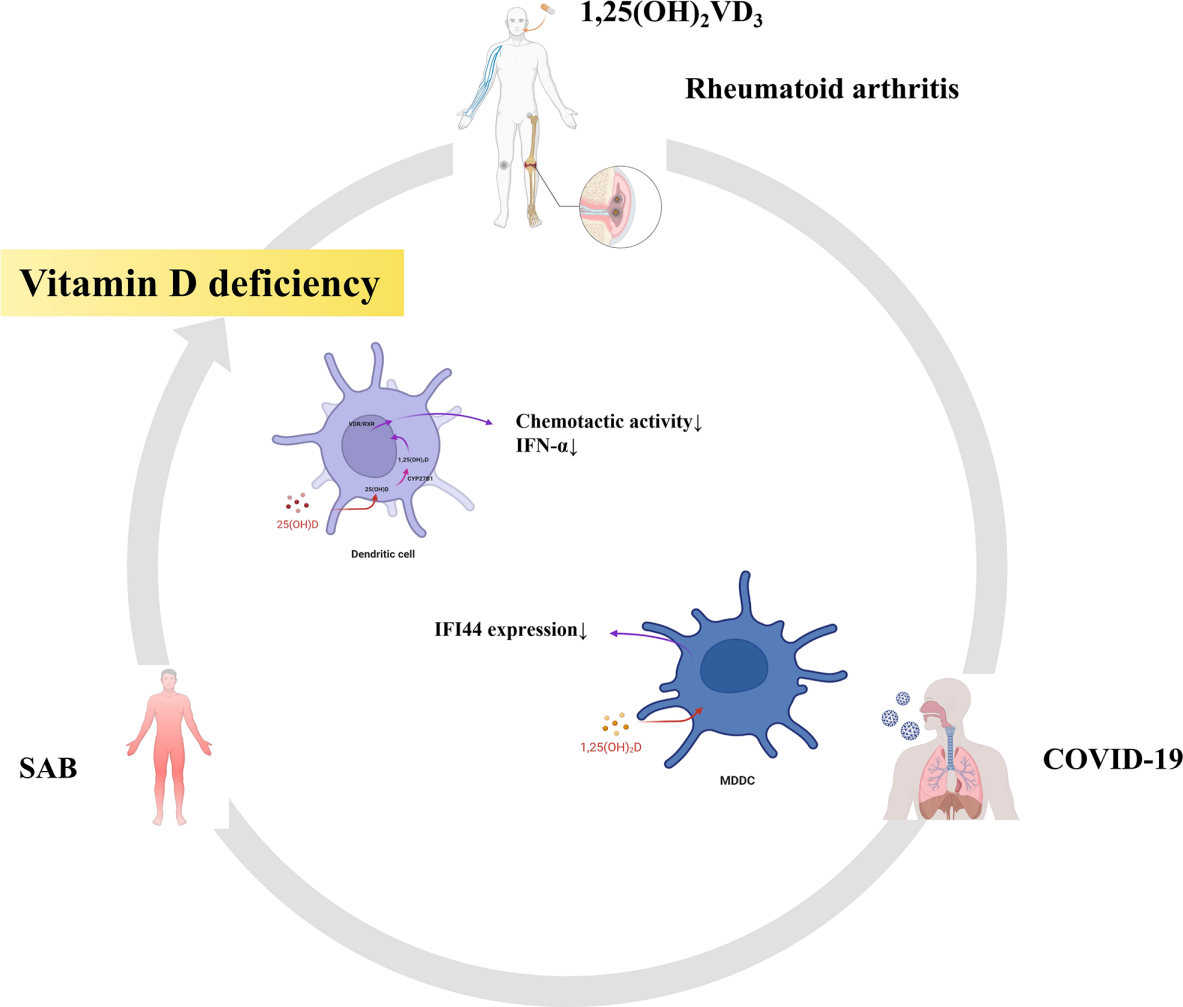
RA, SAB, and COVID-19 are often associated with vitamin D deficiency. This diagram shows that 1,25(OH)_2_VD_3_ is the common target drug for RA, SAB, COVID-19, IFI44, and dendritic cells.

Finally, we also found that part of the pathway of the top 10 hub genes of RA and SAB was enriched in non-genomic actions of 1,25-dihydroxy vitamin D_3_ and that IFI44 was positively correlated with DCs in an immune infiltration correlation analysis in RA. We put 1,25(OH)_2_VD_3_ in series with RA, *S. aureus* infection, COVID-19, IFI44, and DCs (**Figure 14**). First, in RA, 1,25(OH)_2_VD_3_ insufficiency is commonly reported among patients with RA (269–271). In a meta-analysis of 24 studies, 1,25(OH)_2_VD_3_ was found to be inversely linked with RA disease activity (272), and the degree of deficiency was utilized as an indication of RA progression (273). Second, in SAB, 1,25(OH)_2_VD_3_ was able to prevent the invasion of *S. aureus* by boosting the expression of mature macrophages, upregulating macrophage complement receptor immunoglobulin (CRIg), and encouraging macrophage phagocytosis (274, 275). Studies have shown that 1,25(OH)_2_VD_3_ levels are significantly lower in *S. aureus*-infected populations than in non–*S. aureus*–infected populations (276), and 1,25(OH)_2_VD_3_ analogs reduce the incidence of PJI in *S. aureus* infections (277, 278). Third, in COVID-19, according to a study conducted in Israel, 1,25(OH)_2_VD_3_ levels were adversely correlated with COVID-19 (279), and COVID-19 populations were frequently associated with vitamin D deficiency (280–282). 1,25(OH)_2_VD_3_ insufficiency is positively associated with the severity and complications of COVID-19 and increases the chance of SARS-CoV-2 infection (283–286). The main reason for this is the ability to inhibit the cytokine storm and excessive inflammatory response in COVID-19 (287); thus, vitamin D can play a role in the prevention (288, 289), mitigation (285, 290), and treatment (291, 292) of COVID-19 (293). Fourth, in IFI44, the addition of 1,25(OH)_2_VD_3_ to MDDCs in autoimmune diseases (SLE) resulted in a 34% reduction in IFI44 expression and the concentration of 1,25(OH)_2_VD_3_ was negatively correlated with the activity of MDDCs in SLE (294). In our study, the expression of IFI44 was found to be positively correlated with DCs, so 1,25(OH)_2_VD_3_ may also have some correlation with DCs. Fifth, in DCs, it was discovered that 1,25(OH)_2_VD_3_ and its analogs inhibited DC chemotactic activity and IFN-α production, which decreased the expression of ISGs (295, 296). In addition, it has also been shown that DCs are potential target cells of 1,25(OH)_2_VD_3_ for RA inhibition (297). Therefore, in this study, 1,25(OH)_2_VD_3_ was found to be a drug target through the enrichment pathway of the shared genes of RA and SAB, and 1,25(OH)_2_VD_3_ was found to be negatively associated with the expression of RA, COVID-19, SAB, IFI44, and the production and chemotactic activity of IFN-α in DCs from a new perspective.

## Conclusions

In our present study, we screened the shared DEGs based on two datasets of RA (GSE93272) and SAB (GSE33341) and identified pathways associated with immunity and viral infection by multi-platform functional enrichment analysis. The following intersections were taken with the COVID-19 gene library to obtain hub genes, and functional enrichment analysis was performed to validate the pathway linkage of hub genes associated with RA, COVID-19, and SAB. The biomarker and disease target shared by RA, COVID-19, and SAB were validated and identified as IFI144 by GSE17755, GSE55235, and GSE13670 datasets. IFI44, a negative regulator of the IFN signaling pathway, promotes viral replication and bacterial proliferation and is an important molecular target for SARS-CoV-2 and *S. aureus* immune escape in RA. DC activation was positively correlated with the expression of IFI44. 1,25(OH)_2_VD_3_ may be an important therapeutic agent in treating RA with SARS-CoV-2 and *S. aureus* infections. Our research can provide new directions for further analysis of its pathogenesis and targeted development of clinical treatments.

## Data availability statement

The datasets presented in this study can be found in online repositories. The names of the repository/repositories and accession number(s) can be found in the article/supplementary material.

## Author contributions

QZ analyzed and wrote the manuscript. DW designed the experiments and analyzed the data. WW devised the concept and supervised the study. All authors contributed to the article and approved the submitted version.

## Acknowledgments

We acknowledge the GEO and Genecards databases for providing their platforms and contributors for uploading meaningful datasets.

## Conflict of interest

The authors declare that the research was conducted in the absence of any commercial or financial relationships that could be construed as a potential conflict of interest.

## Publisher’s note

All claims expressed in this article are solely those of the authors and do not necessarily represent those of their affiliated organizations, or those of the publisher, the editors and the reviewers. Any product that may be evaluated in this article, or claim that may be made by its manufacturer, is not guaranteed or endorsed by the publisher.

## Glossary

**Table:**

Go	Gene Ontology
KEGG	Kyoto Encyclopedia of Genes and Genomes
PPI	Protein-Protein Interaction
TF	Transcription Factor
ROC	Receiver Operating Characteristic Curve
NLR	NOD-Like Receptor
RSAD2	Radical S-adenosyl Methionine Domain-containing Protein 2
IFIT3	Interferon Induced Protein with Tetratricopeptide Repeats 3
GBP1	Guanylate-Binding Protein 1
RTP4	Receptor Transporter Protein 4
IFI44	Interferon-Induced Protein 44
OAS1	2',5'-Oligoadenylate Synthetase 1
IFI44L	Interferon-induced Protein 44-Like
ISG15	Interferon-stimulated Gene 15
HERC5	HECT Domain and RCC1-Like Domain-Containing Protein 5
IFIT5	Interferon-Induced Protein with tetratricopeptide repeats 5
HC	Healthy Controls
NOD	Nucleotide-Binding Oligomerization Domain
AUC	Area Under the Curve
STAT	Signal Transducer and Activator of Transcription
IRF-3	Interferon Regulatory Factor 3
NF-κB	Nuclear Factor-kappa B
TBK-1	TANK-binding Kinase 1
cGAS	Cyclic GMP-AMP Synthase
IκBs	IκB proteins
ISGF3	Interferon-Stimulted Gene Factor 3
AID	Autoimmune Disease
TLR	Toll-Like Receptor
RLR	RIG-I-Like Receptor
SOCS	Suppressors of Cytokine Signaling
USP18	Ubiquitin-Specific Peptidase 18
SeV	Sendai Virus
LCMV	Lymphocytic Choriomeningitis Virus
VSV	Vesicular Stomatitis Virus
IAV	Influenza A Virus
SLE	Systemic Lupus Erythematosus
